# Exposure to HIV-1 Directly Impairs Mucosal Epithelial Barrier Integrity Allowing Microbial Translocation

**DOI:** 10.1371/journal.ppat.1000852

**Published:** 2010-04-08

**Authors:** Aisha Nazli, Olivia Chan, Wendy N. Dobson-Belaire, Michel Ouellet, Michel J. Tremblay, Scott D. Gray-Owen, A. Larry Arsenault, Charu Kaushic

**Affiliations:** 1 Center For Gene Therapeutics, Michael G. DeGroote Center for Learning and Discovery, McMaster University, Hamilton, Ontario, Canada; 2 Department of Pathology and Molecular Medicine, McMaster University, Hamilton, Ontario, Canada; 3 Department of Molecular Genetics, University of Toronto, Medical Sciences Building, Toronto, Ontario, Canada; 4 Department of Medical Biology, Laval University, Quebec City, Quebec, Canada; Northwestern University, United States of America

## Abstract

While several clinical studies have shown that HIV-1 infection is associated with increased permeability of the intestinal tract, there is very little understanding of the mechanisms underlying HIV-induced impairment of mucosal barriers. Here we demonstrate that exposure to HIV-1 can directly breach the integrity of mucosal epithelial barrier, allowing translocation of virus and bacteria. Purified primary epithelial cells (EC) isolated from female genital tract and T84 intestinal cell line were grown to form polarized, confluent monolayers and exposed to HIV-1. HIV-1 X4 and R5 tropic laboratory strains and clinical isolates were seen to reduce transepithelial resistance (TER), a measure of monolayer integrity, by 30–60% following exposure for 24 hours, without affecting viability of cells. The decrease in TER correlated with disruption of tight junction proteins (claudin 1, 2, 4, occludin and ZO-1) and increased permeability. Treatment of ECs with HIV envelope protein gp120, but not HIV tat, also resulted in impairment of barrier function. Neutralization of gp120 significantly abrogated the effect of HIV. No changes to the barrier function were observed when ECs were exposed to Env defective mutant of HIV. Significant upregulation of inflammatory cytokines, including TNF-α, were seen in both intestinal and genital epithelial cells following exposure to HIV-1. Neutralization of TNF-α reversed the reduction in TERs. The disruption in barrier functions was associated with viral and bacterial translocation across the epithelial monolayers. Collectively, our data shows that mucosal epithelial cells respond directly to envelope glycoprotein of HIV-1 by upregulating inflammatory cytokines that lead to impairment of barrier functions. The increased permeability could be responsible for small but significant crossing of mucosal epithelium by virus and bacteria present in the lumen of mucosa. This mechanism could be particularly relevant to mucosal transmission of HIV-1 as well as immune activation seen in HIV-1 infected individuals.

## Author Summary

Clinical studies have shown that HIV-1 infected patients have increased intestinal permeability. In chronically infected patients that progress to AIDS, there is activation of immune cells consistent with leakage of microbes via the gut. However, the mechanism by which this occurs is not clear. Here, we show that direct exposure of intestinal and genital epithelial cells to HIV leads to breaching of the mucosal barrier and increased leakage of both bacteria and virus across the epithelium. The mechanism of this breakdown appears to be due to inflammatory factors produced by epithelial cells themselves, in response to HIV-1 exposure, that destroy the tight junctions between epithelial cells, thereby allowing microbes access to the inside of the body. Interestingly, we found that treatment of epithelial cells with just the surface glycoprotein from HIV could lead to similar breakdown of the barrier. This implies that when mucosal epithelial cells come in direct contact with large amounts of HIV-1, the virus can cross into the inside of the body and cause direct infection of target cells. The crossing of the bacteria by similar mechanism can lead to chronic inflammation and activation of immune cells of the body.

## Introduction

The mucosa presents a primary barrier against a multitude of micro-organisms present on the mucosal surfaces of the human body [1]. The intestinal and upper reproductive tract are lined by a continuous monolayer of columnar epithelial cells that is responsible for maintaining the physical and functional barrier to harmful microorganisms, such as bacteria and their products, including bacterial toxins as well as commensal organisms [2]–[4]. The preservation of the barrier function is dependent on the intactness of apical plasma membrane on the epithelial cells as well as the intercellular tight junctions. The disruption of the tight junctions can cause increased permeability, leading to “leakiness” such that normally excluded molecules can cross the mucosal epithelium by paracellular permeation, and could lead to inflammatory conditions in the mucosa.

Various pathogenic organisms have developed strategies to either infect or traverse through the epithelial cells at mucosal surfaces, as part of the strategy to establish infection in the host. In fact, mucosal transmission account for majority of infections in humans [5]. Viruses such as rotavirus and astrovirus as well as bacteria such as enteropathogenic *E. Coli* and *C. difficile* are known to increase intestinal permeability by disrupting tight junctions, as part of their pathogenesis [6]–[9]. Increased permeability is also related to a number of other disease conditions that may or may not be related to infection by a pathogen. Crohn's disease, a chronic inflammatory condition of the intestines is characterized by defective tight junction barrier functions, manifested by increased intestinal permeability, although the etiology of the disease is not clearly understood [10].

HIV-1 infection is initiated primarily on mucosal surfaces, through heterosexual or homosexual transmission [1],[11]. In fact, mucosal transmission accounts for greater than 90% of HIV infection [12],[13]. A number of clinical studies have reported intestinal barrier dysfunction, especially during chronic stage of HIV infection [14]–[19]. However, the pathophysiologic mechanism associated with compromised barrier function and whether HIV-1 plays a direct role in this is still unclear. Currently, epithelial barrier defect during HIV-1 infection is thought to be a consequence of mucosal T cell activation following infection, which could lead to increased production of inflammatory cytokines [19],[20]. The intestinal barrier dysfunction has also been implicated as the cause of systemic immune activation during chronic phase of HIV infection, although a recent study has raised the possibility that this may not be universal phenomenon [20],[21]. Studies that have demonstrated immune activation propose this to be the main driving force for progressive immune failure leading to the immunodeficiency stage [22]–[24]. In these studies, HIV disease progression was shown to correlate with increased circulating level of LPS, considered an indicator of microbial translocation, in chronic HIV-infected individuals [25]. Interestingly, immune activation was observed in both the chronic as well as acute phase of HIV infection [25]. The source of or mechanism whereby microbial products could cross the epithelial barrier leading to immune activation have not been elucidated thus far.

In the present study, we investigated the direct effects of HIV-1 exposure on intestinal and genital mucosal epithelia, where primary HIV-1 infection is frequently initiated. We show that in fact the impairment of epithelial barrier function can be a direct result of exposure to HIV-1. Using ex-vivo cultures of pure primary genital epithelium as well as an intestinal epithelial cell line, we show significantly decreased barrier functions and enhanced permeability that is not unique to the intestinal epithelium; similar increase in permeability was seen in the genital epithelium as well. Small amounts of both bacterial and viral translocation were seen following HIV-1 exposure. The mechanism appears to be mediated by increased production of inflammatory cytokines directly from the epithelial cells following exposure to HIV-1, including TNF-alpha, known to disrupt barrier functions. Further, we show that HIV-1 envelope protein gp120 was able to impair barrier functions in epithelial cells on its own. Neutralization of gp120 or exposure to HIV-1 lacking gp160 surface envelope glycoprotein did not have any effect on epithelial cells. These results provide strong evidence that exposure to HIV-1 may lead to impairment in barrier function of mucosal epithelium which could result both in translocation of HIV-1 and/or luminal bacteria that could serve as the source of immune activation during HIV-1 infection.

## Results

### Genital and intestinal epithelial monolayer transepithelial resistances (TER) are decreased following exposure to different strains of HIV-1

**Figure 1:**
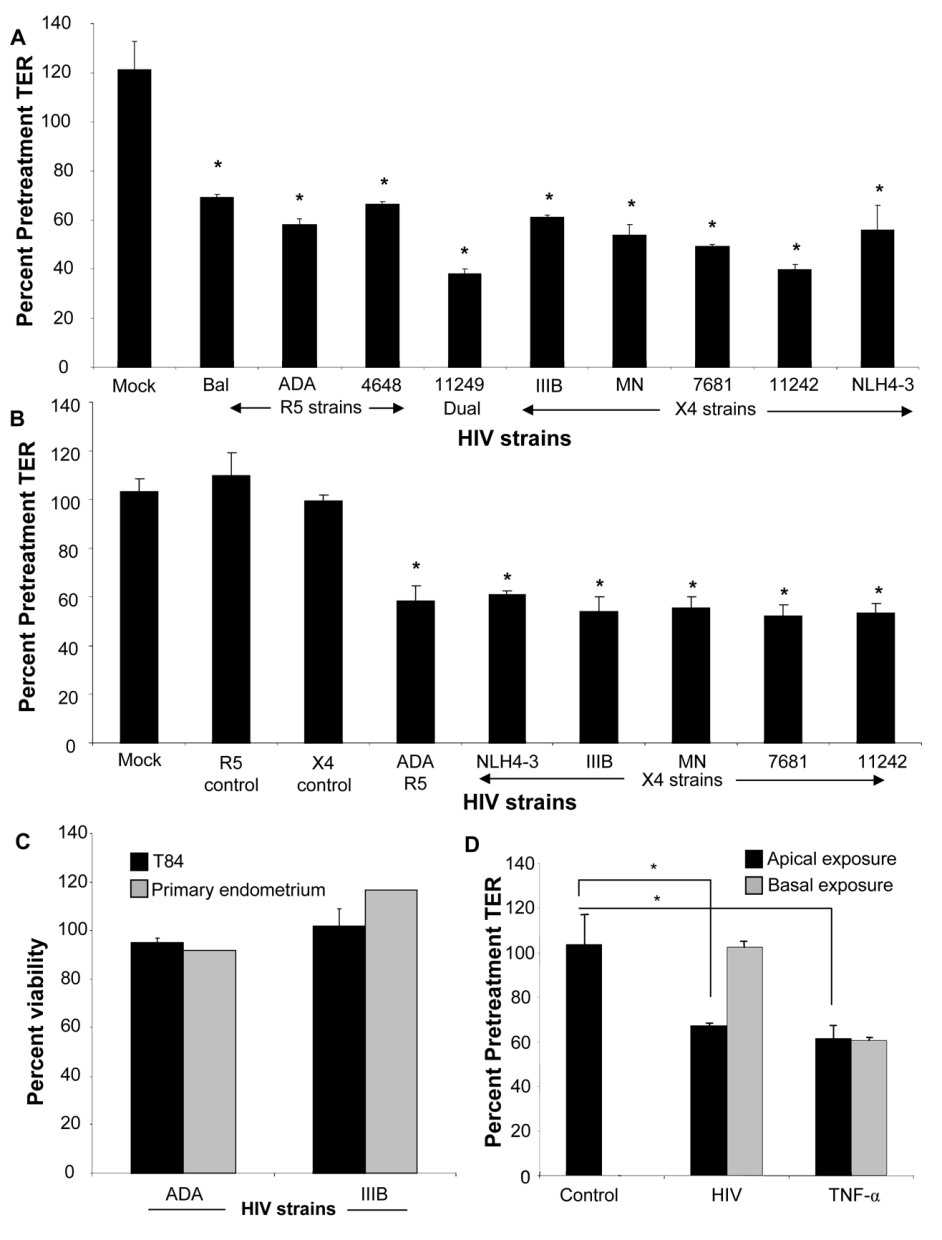
Primary endometrial epithelial monolayers (A) and T84 intestinal epithelial cell line (B) were exposed to 10^6^ infectious viral units/ml of HIV-1 laboratory strains Bal, NL4-3, ADA (R5 tropic) and IIIB, MN (X4 tropic) and four clinical strains 4648 (R5 tropic) 11242, 7681 (X4 tropic) and 11249 (dual tropic). Corresponding p24 values were as follows for R5 tropic: Bal (0.6ng/ml), ADA (two different viral stocks 0.3 ng/ml and 1040 ng/ml), 4648 (49ng/ml); X4 tropic: IIIB (0.7ng/ml and 161ng/ml), MN (103ng/ml and 1110ng/ml), NLH4-3 (93ng/ml), 7681 (773ng/ml), 11242 (342ng/ml); dual tropic 11249 (200ng/ml). Control include cultures that were mock-treated with medium without virus (mock) or virus-free supernatants (R5 and X4 control). Transepithelial resistance (TER), was measured prior to and 24 hours post- exposure to HIV-1. *p<0.001, n = 3–9. Viability of primary female genital epithelial cells and T84 intestinal epithelial cells was assessed by MTT assay 24 hours after exposure with two HIV strains IIIB (X4-tropic) and ADA (R5-tropic) and compared with mock-infected epithelial monolayers (C). The effect of apical and basolateral exposure was determined. (D). Differentiated T84 epithelial monolayers were mock infected or exposed apically or basolaterally to HIV (ADA strain, 10^6^ infectious viral particles/ml). TER was measured at 0, 24 and 48 hours post exposure. *P<0.001, **P<0.0001, n = 3.

In order to study HIV-1 induced barrier defect in epithelial monolayers, HIV-1 (10^6^ infectious viral units/ml) was added apically to confluent monolayers of differentiated primary female genital epithelial cells (ECs) or T84 intestinal epithelial cells grown in transwells. Transepithelial resistance (TER), a measure of epithelial monolayer integrity, was measured before and 24h post-infection and calculated as a percentage of pretreatment TER. Transepithelial resistances of primary endometrial epithelial monolayers exposed to various strains of HIV-1 were significantly reduced (p<0.001 with all HIV-1 strains) by 30–60%, 24h post-exposure (Figure 1A). The TER decrease in primary endometrial epithelial monolayers was more pronounced following exposure to clinical strains of HIV-1 [11242 (60.3±3.16%), 11249 (62.1±3.0%), 4648 (33.3±1.2%), 7681 (50.8±1%)] as compared to laboratory strains (R5 tropic: Bal, ADA; X4 tropic: NLH4-3, IIIB and MN) of HIV-1 (44.1–33.7±1.2–6.3%). Similar TER decrease (39.0–47.8+1.6–6.0%, p<0.001, Figure 1B) was observed in T84 monolayers with an array of laboratory and clinical strains. Controls included mock-treatment of monolayers with same volume of media, without HIV-1 (media control). Additional controls included treating confluent monolayers with virus-free supernatant from cultures used for preparing virus stocks (Figure 1B, R5 and X4 mock control). All mock-infected cultures maintained or showed increase in TERs at 100–120% pretreatment values in both primary endometrial and T84 epithelial cells.

To exclude the possibility that changes in the barrier function resulted from any cytotoxic effect of HIV-1 exposure that would result in breach of the integrity of the monolayers, we tested the viability of both intestinal and endometrial epithelial cultures following HIV-1 exposure by an MTT assay. The results indicated that exposure to HIV-1 for 24h did not have any affect on cell viability relative to controls (Figure 1C). Thus reduction in TERs following HIV-1 exposure in both intestinal and genital epithelial cultures was not due to compromised viability of epithelial cells.

We also determined if exposure to HIV on the basolateral side of the epithelial cell monolayer decreased TERs of epithelial monolayers. T84 intestinal EC monolayers were exposed to HIV on both apical and basolateral surface and TERs were compared 24 hours later. Basolateral exposure to HIV led to no significant decrease in TER (Figure 1D). This lack of response to basolateral stimulation appeared to be specific for HIV-1, since exposure to TNF-α decreased TERs of confluent epithelial monolayers, regardless of whether the stimulation was delivered on apical or basolateral surface of cells (Figure 1D).

### HIV-1 exposure down-regulates tight junction mRNA and protein levels in epithelial monolayers

**Figure 2:**
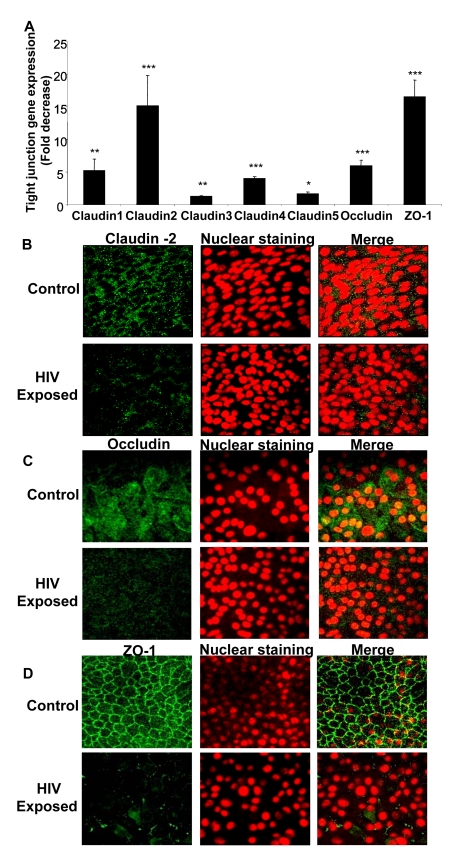
Confluent monolayers grown from primary endometrial epithelial cells were either mock-treated or exposed to HIV-1 (ADA strain, 10^6^ infectious viral units/ml, p24 280ng/ml) for 8 hours. Total RNA was extracted and cDNA was synthesized. Quantitative Real-time RT-PCR was conducted for tight junction gene expression by measuring mRNA for Claudin 1–5, Occludin and ZO-1. GAPDH, a house keeping gene, was measured for internal control (A). * p<0.01; ** p<0.001; *** p<0.0001). Immunofluorescent staining of tight junction proteins following HIV-1 exposure compared to mock-treated epithelial monolayers. Representative staining is shown for claudin-2 (B), Occludin (C), and ZO-1 (D) at 24 hours post-exposure. Magnification: 1260×. Data shown is representative of 3 separate experiments, each experiment had 3–5 replicate cultures for each experimental condition. For RNA extraction, 6–8 replicate cultures were pooled.

We next examined the effect of HIV-1 exposure on gene and protein expression of tight junctions in genital and intestinal epithelial monolayers. Confluent monolayers of primary endometrial epithelial cells were mock-treated or exposed to HIV-1 for 8h and total mRNA was extracted and subjected to quantitative real time RT-PCR using primers specific to different tight junction genes including Claudin 1, 2, 3, 4, 5, Occludin and ZO-1 (Table 1). The expression of all seven junction genes that were examined was down-regulated 2–17 fold in HIV-1 exposed monolayers when compared to mock-treated controls (Figure 2A). Claudin-1, Claudin-2, Claudin-3, Claudin-4, Claudin-5, Occludin and ZO-1 all showed significantly lower expression (P<0.0001–0.01). Similar decrease in mRNA expression was seen with intestinal epithelial monolayers exposed to HIV-1 (data not shown).

**Table 1:** Primers used for Real Time PCR for different tight junction genes.

Tight junction genes	Primer names	Primer sequence 5′ - 3′	References
ZO-1	ZO-1F	TGTGAGTCCTTCAGCTGTGGAA	Pu H, Tian J, Andras AS, Hayashi IK, et al, (2005) HIV-1 Tat protein-induced alterations of ZO-1 expression are mediated by redox-regulated ERK 1/2 activation. J Cerebral Blood Flow & Metabolism 25:1325–1335
	ZO-1R	GGAACTCAACACACCATTG	
Occludin	Occlu F	CATTGCCATCTTTGCCTGTG	Bai L, Zhang Z, Zhang H, et al. (2008) HIV-1 Tat protein alter the tight junction integrity and function of retinal pigment epithelium: an in vitro study. BMC Infec. Dis..8:77–89
	Occlu R	AGCCATAACCATAGCCATAGC	
Claudin 1	Claudin 1F	CAGGCTACGACCCGAAC	Bai L, et al (2008) BMC Infec. Dis..8:77–89
	Claudin 1R	CAGGCTACGCAAGGA	
Claudin 2	Claudin 2F	CCCAAACCCACTAATCACATC	Bai L. et al (2008) BMC Infec. Dis..8:77–89
	Claudin 2R	GCCACTGCTTCTCCTTCC	
Claudin 3	Claudin 3F	CAGGCTACGACCGCAAGGAC	Bai L. et al (2008) BMC Infec. Dis..8:77–89
	Claudin 3R	GGTGGTGGTGGTGGTGTTGG	
Claudin 4	Claudin 4F	GGCGTGGTGTTCCTGTTG	Bai L. et al (2008) BMC Infec. Dis..8:77–89
	Claudin 4R	AGCGGATTGTAGAAGTCTTGG	
Claudin 5	Claudin 5F	TACCGCAGGAAGAGGAGCAG	Bai L. et al (2008) BMC Infec. Dis..8:77–89
	Claudin 5R	GCCCGAAGCAGCCAATCC	
GAPDH	GAPDH F	TCTCTGCTCCTCCTGTTC	Bai L. et al (2008) BMC Infec. Dis..8:77–89
	GAPDH R	CTCCGACCTTCACCTTCC	

To correlate the decreased mRNA levels with tight junction protein expression, intestinal and endometrial monolayers were stained for different tight junction proteins 24h after HIV-1 exposure and compared with mock-treated monolayers by confocal microscopy. The protein expression correlated well with real-time quantitative RT-PCR results, showing distinct decrease in localization of Claudin 2 and Occludin and ZO-1 (Figure 2B–D). The disruption of ZO-1 localization in HIV-1 exposed monolayers was particularly severe and clearly visible. These results indicated overall disruption of tight junctions.

### HIV-1 mediated decrease in TERs correlates with increased leakage of Blue Dextran

**Figure 3:**
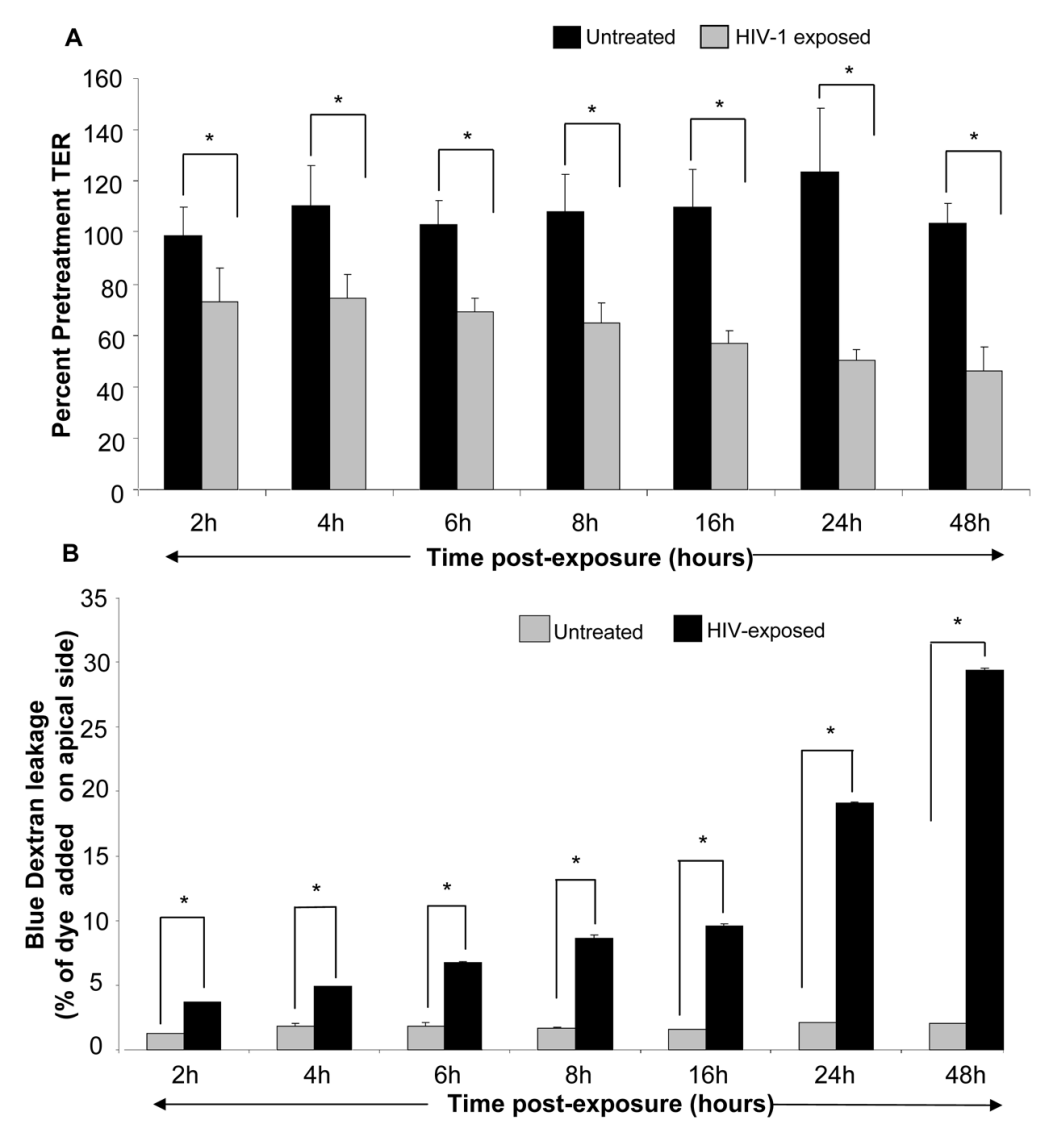
Primary genital epithelial monolayers were exposed to HIV-ADA (R5 strain, 10^6^ infectious viral units/ml, p24 280ng/ml ) for 2h, 4h, 6h, 8h, 16h, 24h and 48h. To measure barrier functions, TER was measured prior to and post exposure, ZO-1 staining was done and Dextran Blue dye leakage was measured across the monolayers at all time points. (A) TER values. p<0.001. (B) Paracellular permeability measured by addition of Blue Dextran dye on the apical side of monolayers. At different time intervals post-exposure, basolateral supernatants were sampled and absorbance was measured and compared to apical absorbance at initial time point (Time “0”). Blue Dextran leakage was calculated as a percentage of apical values. Data shown is representative of 3–4 separate experiments, each experiment had 3–5 replicate cultures for each experimental condition.

**Figure 4:**
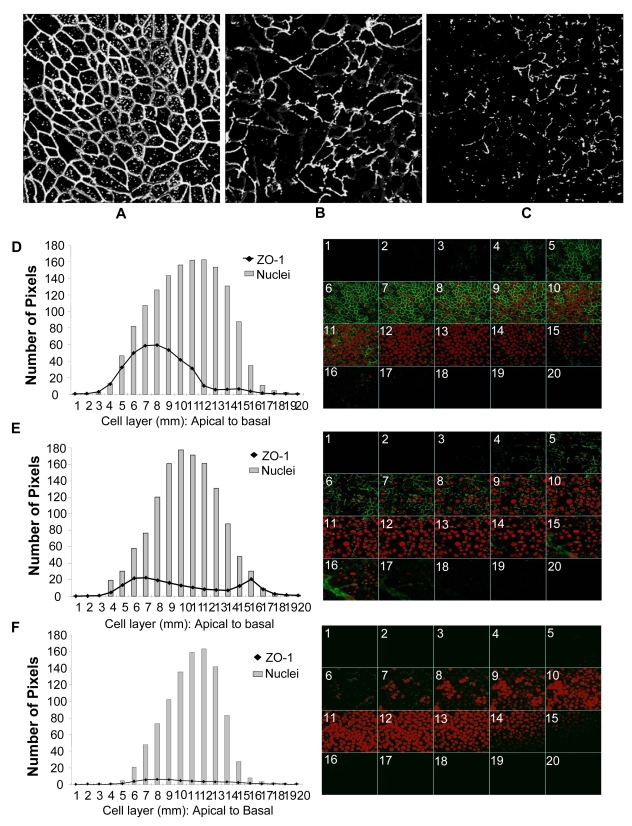
Genital EC monolayers were fixed after 4 or 24 hours post-HIV-1 exposure and stained for ZO-1 (green) and nucleus (red). A–C. Series of stack planes (XY) taken through the apical extent of the monolayer. D–F. Quantification of ZO-1 (dark line) and nuclear (grey bars) staining shown as graphs. Each cell layer (1–20) corresponds to series of images from Z-stack sections taken at 1µm thickness through the cell monolayer shown on the right. X-axis illustrates cell layers from apical to basolateral. Y-axis illustrates the number of pixels present over the entire area of image. A, D. Control mock infected monolayer, 24 hours post-treatment. B,E. HIV-1 exposed monolayer, 4 hours post-treatment. C,F. HIV-1 exposed monolayer, 24 hours post-treatment. Results shown are representative of 3 separate Z-stacks collected and analyzed from each replicate, each treatment group had 3–5 replicates and the experiment was repeated 3 times. (Magnification :1260×).

Next, we conducted a time course study to determine if HIV-1 mediated decrease in TER correlated with alterations in permeability of the monolayers. Polarized, confluent monolayers of primary endometrial and T84 intestinal epithelial monolayers were exposed to HIV-1 for different lengths of time. The TERs were measured prior to treatment and at 2h, 4h, 6h, 8h, 16h, 24h and 48h post-exposure. A reduction in TER was first evident after 2h of HIV-1 exposure of genital epithelial cells (Figure 3A) (p<0.001). TERs continued to decrease with longer exposure times, reaching a low of ∼40% of pre-treatment value at 24–48hours. The TERs of control mock-treated group remained relatively unchanged or showed slight increase through the duration of the experiment (Figure 4A).

The decrease in TER was correlated with leakage of Blue Dextran dye into basolateral compartment. Blue Dextran dye (mol. wt. 2000 kDa), added to the apical side of intact epithelial monolayers, normally cannot pass through the tight junctions between epithelial cells that prevent its paracellular transport [26]. A disruption of tight junctions allows leakage of this dye to the basolateral compartment which can be detected by densitometric measurement. To determine if HIV-1 exposure led to increased permeability, Blue Dextran dye was added to mock-treated control as well as HIV-1 (ADA) exposed monolayers on apical side and its leakage was detected in basolateral compartment after different time intervals. Blue Dextran leakage became evident 2h post-exposure (p<0.01) and gradually increased with increased exposure time (Figure 3B). The kinetics of Blue Dextran accumulation in the basolateral compartment closely paralleled the decrease in the TERs post-exposure.

### Disruption of tight junctions is due to de-localization of ZO-1 at earlier time points followed by decrease in ZO-1 protein

Among the tight junction proteins, the normal pattern of ZO-1 localization was very prominent in both primary genital ECs and intestinal T84 cells. Equally striking and apparent was its disruption following HIV-1 exposure. Therefore, ZO-1 was localized and quantified in confluent EC monolayers post-HIV-1 treatment and compared with control cultures to determine the disruption of tight junctions. The results are shown for an earlier time point (4 hour) and a later time point (24 hour) in control and HIV-1 exposed monolayers (Figure 4). Confocal scanning was performed and images were captured as Z-stacks to ensure that the entire depth of the cell monolayer was encompassed. Control monolayers were characterized by well-defined, lacey and interconnected ZO-1 staining pattern located at the perimeter of each cell (Figure 4 A and D) that remained unchanged over time. Punctate distribution of intracellular ZO-1 was also commonly noted in confluent ECs. Quantitative analysis of the ZO-1 (green) and nuclear (red) fluorescence in the Z-stacks indicated that ZO-1 staining was localized in the apical cell layers (5–10µm) of the EC, with the peak signal observed in cell layers 7–8µm, while the peak of nuclear staining was more towards the center of the cells (cell layers 10–12µm). Four hours following HIV-1 exposure there was a marked decrease in ZO-1 signal (Figure 4E). Even more remarkable was the clear disruption in the ZO-1 localization, seen as discontinuous distribution pattern around the perimeter of cells. The inter-nodal connections between cells, indicating intact tight junctions, were also notably decreased (17 in HIV-1-treated versus 203 in control monolayer). Interestingly, the signal for ZO-1 staining was present not just in the apical layers but in basal cell layers as well, indicating a displacement of ZO-1 protein from the tight junctions. The reduction in ZO-1 signal was progressive and analysis of 24 hours post-treatment cultures indicated virtual absence of ZO-1 signal (Figure 4 C and F), consistent with decreased transcription of the mRNA seen at 8 hours post-treatment (Figure 2A).

### The decrease in TER and reduction in tight junction staining is related to virus concentration

**Figure 5:**
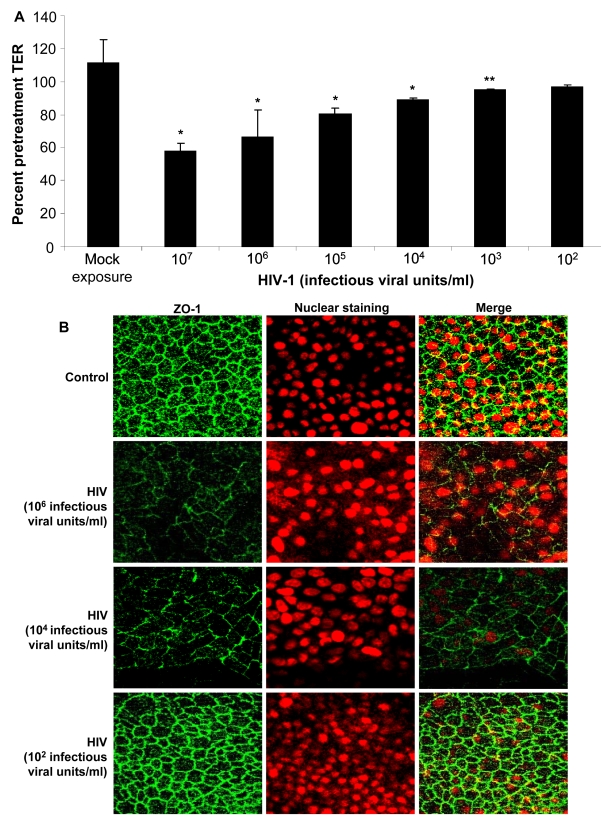
Primary endometrial EC monolayers were exposed to different concentrations of HIV-1 (ADA) for 24 hours. TER values were measured, starting at viral concentration of 10^3^ up to 10^7^ infectious viral units/ml (equivalent to p24 values of 0.2ng–2800 ng/ml) (A).* p<0.001 **p<0.05. ZO-1 staining following exposure to different concentration of virus (B). Data shown is representative of three separate experiments, each experiment had 3–5 replicate cultures for each experimental condition.

Next we determined whether the impairment of barrier function of the epithelium was dependent on the exposure dose of HIV. Various concentrations of HIV-1 strain ADA (10^2^–10^7^ infectious viral units/ml corresponding with MOI of 1∶10^−4^ to 1∶10 and p24 values of 0.2ng–2800ng/ml) were added on apical side of confluent EC monolayers. The TER values were measured 24h post-exposure and expressed as percent pretreatment TER (Figure 5A). Exposure to HIV-1 concentrations from 10^7^–10^3^ infectious viral units/ml showed significant reduction in TER compared with uninfected controls (P<0.001, 10^7^–10^4^ infectious virus units/ml; P<0.05, 10^3^ infectious virus units/ml). Exposure to the lowest concentration corresponding to MOI of 1∶10^−4^ (p24 value of 0.2ng/ml) did not cause any significant decrease in TER. To confirm that decreased TER corresponded with disruption in tight junctions, epithelial monolayers exposed to various concentrations of HIV-1 were stained for ZO-1. The ZO-1 disruption was clearly visible at higher exposure doses of HIV-1 (Figure 5B).

### HIV-1 induced barrier permeability is independent of viral replication

**Figure 6:**
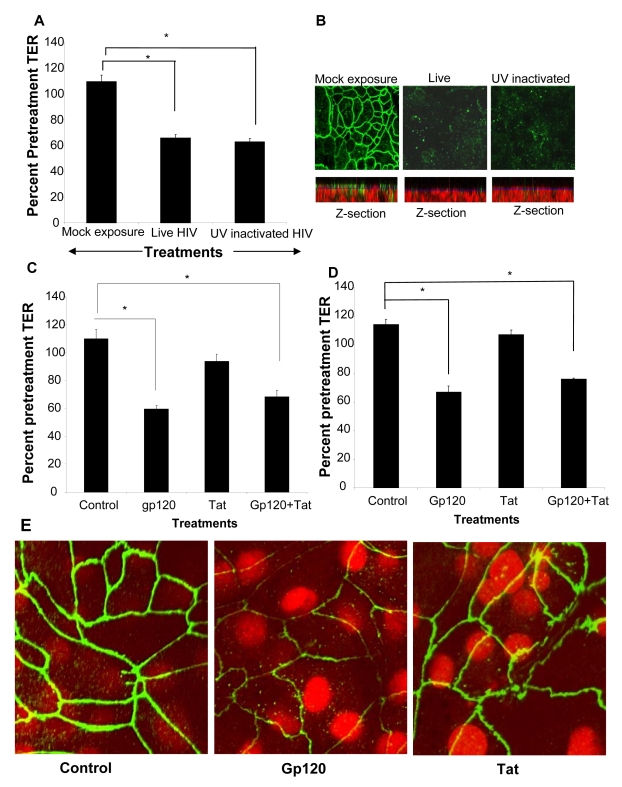
HIV-1 induced barrier permeability is independent of viral replication. Differentiated T84 cells were mock infected or exposed to live or UV inactivated HIV (IIIB strain, 10^6^ infectious viral particles/ml). (A) TER values following exposure to both live and UV-inactivated HIV compared to mock treated monolayer (P<0.001). (B) ZO-1 staining in epithelial cells exposed to live and UV-inactivated HIV, but not mock infected cultures. The corresponding Z-stack series below each panel clearly shows majority of ZO-1 staining (green) in mock treated cultures on the apical side of the monolayer, while the nuclei are seen more basolaterally (red). Magnification:1260×. Primary endometrial EC monolayers (C) or intestinal T84 cells (D) were treated with either gp120 (0.1µg/ml, 0.8nM) or Tat (1.4ug/ml, 100nM) or a combination of both, for 24 hours. TER values were measured prior to and post-treatment. p<0.001, n = 6. (E) ZO-1 staining after gp120 treatment and Tat treatment. Magnification: 2520×. Data shown is representative of two (A,B) and six (C,D) separate experiments, each experiment had 3–5 replicate cultures for each experimental condition.

To determine if productive viral replication was required for increased permeability, polarized T84 cells were treated apically with infectious HIV or an equivalent amount of UV-inactivated virus (Figure 6A). Monolayers treated with UV-inactivated HIV demonstrated decrease in TER nearly identical to those of the live, infectious virus (p<0.001). The disruption of ZO-1 in epithelial cell monolayers treated with UV inactivated was similar to that seen with live HIV (Figure 6B). This was also clearly visible in the z-axis reconstructions where mock-treated monolayers showed abundant ZO-1 staining on the apical lateral membranes which was virtually absent in EC monolayers exposed to live and UV inactivated HIV-1 (Figure 6B, Z-section). These results indicated that the increased epithelial cell permeability could be due to initial attachment of the virus. This was further investigated by exposing epithelial cells to HIV surface glycoprotein.

### Gp120 but not Tat treatment compromises epithelial barrier function

Previous studies examining effect of HIV-1 on blood brain barrier demonstrated that HIV-1 gp120, a surface envelope glycoprotein and tat, an HIV regulatory protein that is produced by infected cells, could directly increase permeability of endothelial cells [27]–[29]. We therefore determined if either of these two viral proteins could exert similar effects on epithelial cell barrier function. Transepithelial resistances were significantly reduced following treatment with gp120 alone or in combination with tat (P<0.001). Tat alone did not have any effect on barrier functions compared with untreated controls. The results were comparable in primary endometrial (Figure 6C) and in T84 intestinal epithelial cells (Figure 6D). Disruption of tight junctions by gp120 was confirmed by ZO-1 staining while tat-treated epithelial monolayers did not show any alterations in ZO-1 localization (Figure 6E).

### HIV-1 induced barrier permeability is dependent on virus envelope glycoprotein

**Figure 7:**
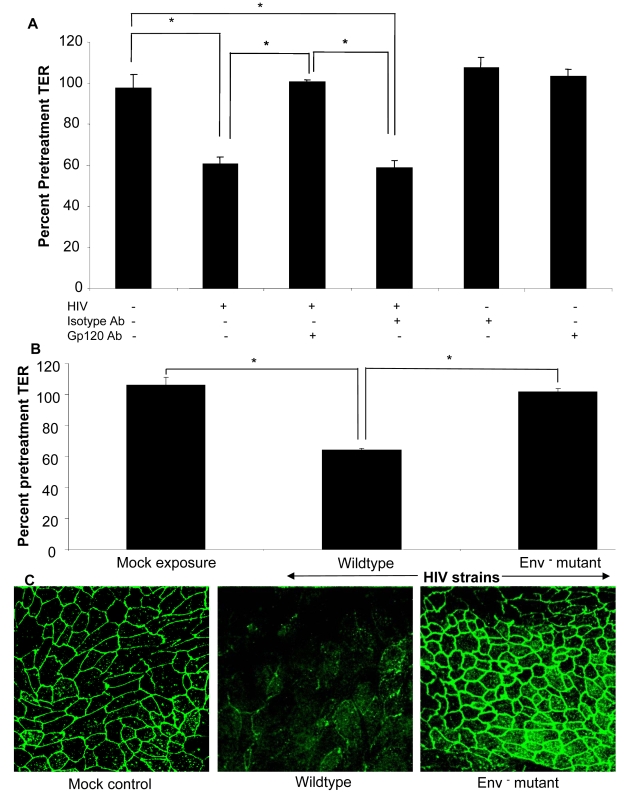
(A) Epithelial monolayers were treated with medium alone, HIV-1 (IIIB, 10^6^ infectious viral particles/ml), HIV-1 in combination with gp120 neutralizing antibody (35µg/ml) or isotype control antibody (35µg/ml), gp120 or isotype antibody alone. TER measurements were taken as a measure of change in permeability and presented as percent of pre-treatment TER. p<0.01. (B) Confluent T84 epithelial cell cultures were mock infected or exposed to NL4-3 (p24, 79 ng/ml) or NL4-3 Env^−^ mutant (p24, 79 ng/ml) and TER measurements were taken prior to and 24 hours post-exposure. P<0.001 (C) ZO-1 localization after exposure to wildtype HIV-1 NL4-3 or Env^−^ NL4-3 mutant. Data shown is representative of four separate experiments, each experiment had 3–5 replicate cultures for each experimental condition.

To confirm that the barrier defect was mediated by HIV-1 viral envelope glycoprotein, we neutralized gp120 on HIV prior to exposure to epithelial cells (Figure 7A). Epithelial monolayers were treated with HIV-1 or equivalent amount of virus that had been pre-incubated with gp120 neutralizing monoclonal antibody or isotype control. Gp120 neutralizing antibody significantly abrogated the increase in permeability (p<0.01), while similar effect was not seen with isotype control, further confirming the role of gp120 in increased barrier permeability.

The final confirmation that viral surface glycoprotein was critical for changes in epithelial cell permeability was obtained by treating epithelial cells with an Env-defective mutant of HIV (Env^−^) (Figure 7B,C) [30]. This mutant is characterized by lack of Env polyprotein which results in absence of both gp120 and gp41, the complex that initiates attachment and binding of HIV-1 to host cell and subsequent entry. Epithelial cells exposure to Env^−^ HIV showed no changes in TERs compared to wild type HIV (p>0.05), while the wild type HIV showed significant decrease in TERs (p<0.01) (Figure 7B). This was further confirmed by intact ZO-1 staining seen in epithelial monolayers exposed to Env^−^ HIV, similar to mock-treated cells (Figure 7C). Combined with the results from gp120 neutralizing antibody described above, these results indicate that the HIV-1 surface glycoprotein is responsible for disruption of epithelial barrier leading to increased permeability.

### HIV-1 exposure induces inflammatory cytokines in genital and intestinal epithelial monolayers

**Figure 8:**
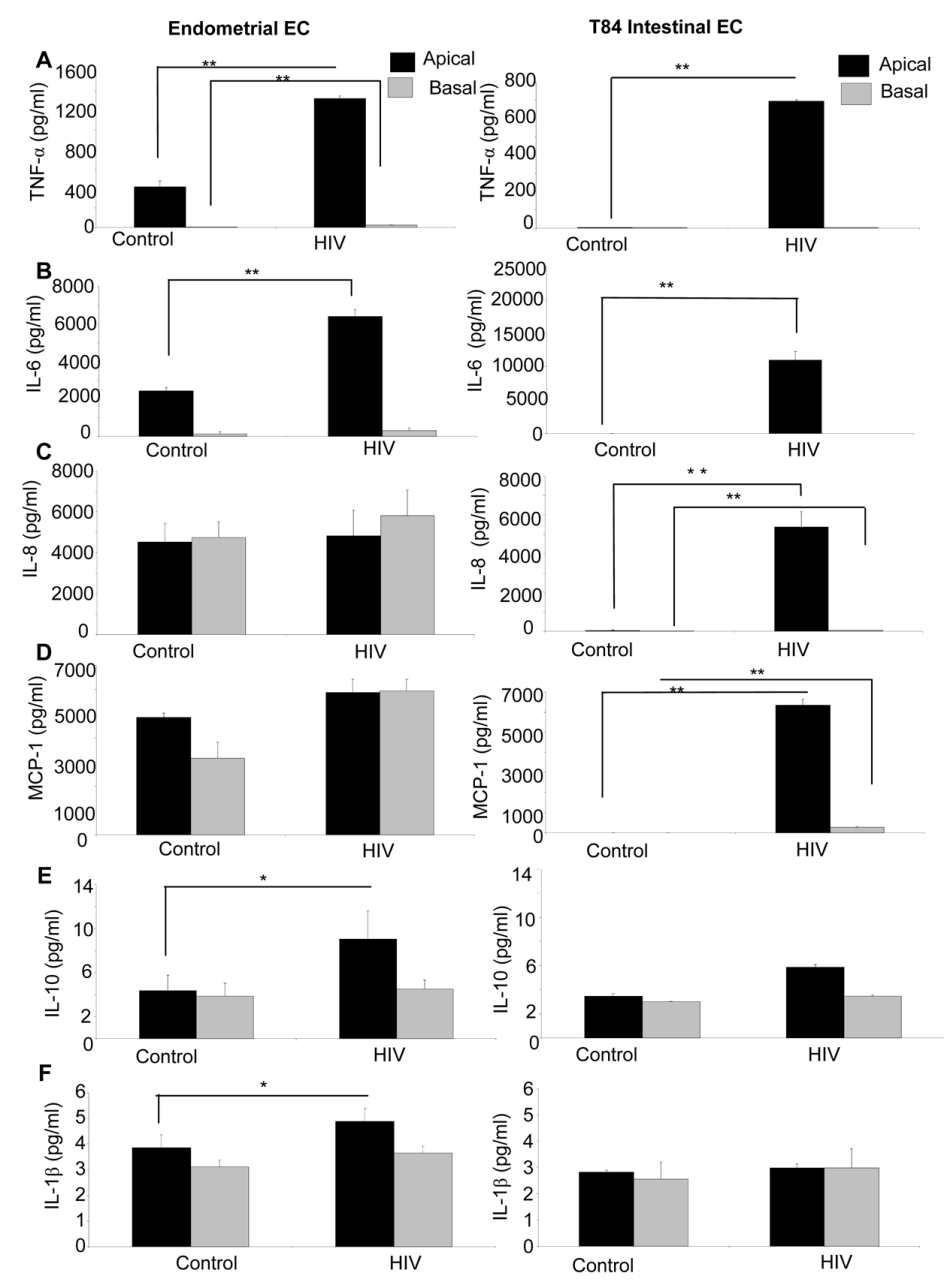
Primary endometrial EC and T84 intestinal monolayers were exposed to HIV-1 (ADA, 10^6^ infectious viral units/ml, p24 280ng/ml) and apical and basolateral supernatants were collected 24 hours post-exposure and assayed by Luminex multi-analyte kit for the following cytokines: (A) TNF-α (B) IL-6, (C) IL-8, (D) MCP-1, (E) IL-10, (F) IL-1β. *p<0.01, **p<0.001. Data shown is representative of three separate experiments from different tissues, each experiment had 3–5 replicate cultures for each experimental condition.

Epithelial cells are known to secrete a variety of cytokines at constitutive levels. Many of these are upregulated or induced *de novo* in response to pathogens such as Neisseria gonorrhea [31]. Additionally, inflammatory cytokines have been shown to mediate enhanced permeability of intestinal epithelial cells [32]. We therefore examined the cytokine secretion profile of epithelial cells following HIV-1 exposure. Apical and basolateral supernatants of genital and intestinal monolayers were collected 24h post HIV-1 exposure and examined for presence of six cytokines known to be secreted by epithelial cells (Figure 8A–F). The T84 intestinal cell line constitutively secreted low levels of IL-10 and IL-1β. In comparison, the primary endometrial epithelial monolayers showed constitutive production of a larger array of cytokines, some of them in high amounts (IL-6, IL-8, MCP-1). Following HIV-1 exposure, there was a significant increase in production of TNF-α, IL-6, IL-8 and MCP-1 in T84 intestinal epithelial monolayers (Figures 8A–D). In primary endometrial ECs there was a significant increase in production of TNF-α, IL-6, MCP-1, IL-10, and IL-1β secretion after 24 hours of HIV-1 exposure (Figures 8A, B, E, F).

### HIV-1 mediated TER decrease is reversed by treatment with anti-TNF antibody

**Figure 9:**
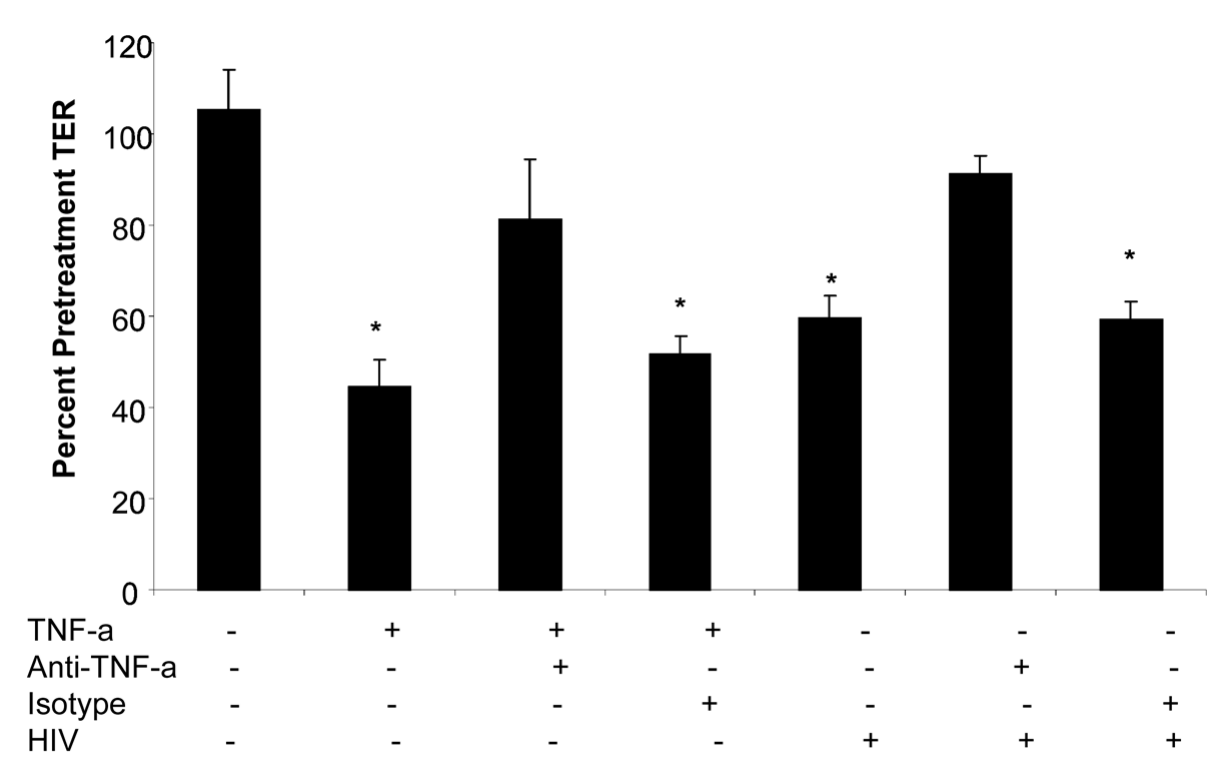
Primary endometrial epithelial monolayers were exposed to TNF-α or HIV-1 alone; TNF-α or HIV-1 (ADA,10^6^ infectious viral units/ml, p24 280ng/ml) in combination with anti-TNF-α neutralizing antibody; TNF-α or HIV-1 in combination with normal mouse serum for 24 hours. TER measurements were taken as a measure of change in permeability and presented as percentage of pre-treatment TER. Data shown is representative of two separate experiments, each experiment had 3–5 replicate cultures for each experimental condition.

Of the cytokines that showed increased production in genital and intestinal epithelial cells following HIV-1 exposure, TNF-α is well known to disrupt epithelial cell tight junction assembly and increase intestinal cell permeability [33]. Since TNF-α was significantly up-regulated following HIV-1 exposure in both genital and intestinal ECs, we neutralized TNF-α to see whether this would affect barrier function alterations. Confluent T84 intestinal epithelial monolayers were treated with TNF-α (20 ng/ml), TNF-α+anti-TNF antibody (mouse anti-human TNF-α , 25 µg/ml), TNF-α+mouse serum (control), HIV-1 alone, HIV-1+anti-TNF antibody (mouse anti-human TNF-α antibody, 25 µg/ml) and HIV-1+mouse serum (control). The TER measurements were taken prior to and 24 hours following the treatments. As expected both TNF-α and HIV-1 caused a significant drop in TER values compared to untreated control monolayers (Figure 9). When epithelial monolayers were pre-treated with anti-TNF-α antibody prior to treatment with TNF-α and HIV-1, the TER values did not decrease significantly over 24 hours of exposure. Incubation of monolayers with normal mouse serum did not show the same effect as the anti-TNF-α antibody. These results provide direct evidence that TNF-α secreted by epithelial cells in response to HIV-1 exposure contributed significantly to the disruption of barrier function in epithelial cells.

### Increased permeability correlates with translocation of virus and bacteria across the epithelial monolayers

**Figure 10:**
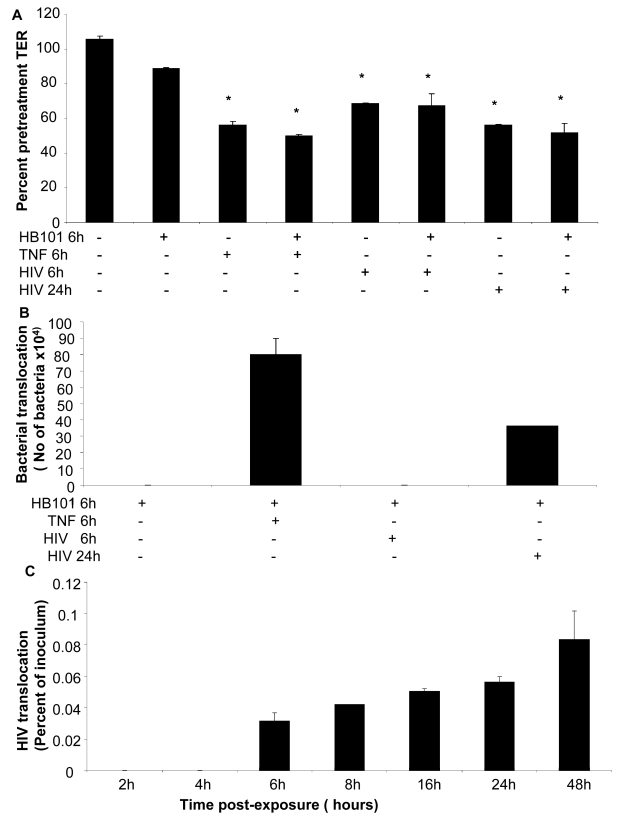
Bacterial and viral translocation across mucosal epithelial monolayers following HIV-1 exposure. (A) Bacterial translocation was measured in T84 intestinal monolayers. Confluent monolayers were left untreated or treated for 6 hours with TNF-α (20ng/ml), *E. coli* (10^8^ CFU/ml) , TNF-α (20ng/ml) +*E. coli* (10^8^ CFU/ml), HIV-1 or HIV-1 (6 or 24 hours)+*E. coli* (10^8^ CFU/ml). (A) TER measurements following various treatments in the presence or absence of E. Coli. * p<0.001. (B) Basolateral supernatants were collected and bacterial counts were done. (C) Viral translocation was determined in endometrial EC monolayers exposed to HIV-1 (ADA, 10^6^ infectious viral units/ml, p24 280ng/ml) on the apical side. Basolateral supernatants were collected after different time intervals infectious and viral counts were done on TZM/b-l indicator cell line. Viral counts are depicted as percentage of inoculum added to the apical compartment of monolayers. Data shown is representative of two separate experiments, each experiment had 3–5 replicate cultures for each experimental condition.

To correlate barrier dysfunction with increased permeability to luminal antigens, we examined bacterial and viral translocation across the epithelial monolayers post-exposure to HIV-1. Intestinal epithelial monolayers grown to confluence were exposed to HIV-1. TNF-α was used as a positive control since it is known to disrupt tight junctions and increase permeability. Because direct exposure to TNF-α for prolonged period of time causes irreversible damage to epithelial cells, TNF-α treatment was limited to 6 hours prior to addition of non-pathogenic *E. Coli* to allow observation of bacterial translocation. Exposure time for HIV-1 was chosen at 6 hours (for comparison with TNF-α) and 24 hours (based on our results of maximum permeability with continued viability). Six hours after addition of *E. Coli* to the apical compartment, basolateral supernatants were collected and plated on LB agar and bacterial colonies were quantified. Transepithelial resistance measured prior to and 24 hours following treatments to determine if addition of *E. Coli* had any effect on TERs (Figure 10A). In HIV-1 exposed monolayers TER decreased significantly within 6h of exposure as expected; further reduction was seen at 24 hours. In comparison, HIV-1 unexposed monolayers only and those that were untreated except with *E. Coli*, TER values were maintained at 106% and 89% percent respectively, of pre-treatment TER values. Bacterial translocation was seen only in monolayers following 24hours of HIV-1 exposure and 6 hours of TNF-α treatment (Figure 10B). Bacterial translocation seen following 24 hours of HIV-1 treatment was about 50% of that seen following 6 hours of TNF treatment. No significant bacterial translocation was seen after 6h of HIV-1 exposure.

In a separate experiment, lipopolysaccaride (LPS) leakage in HIV-1 exposed endometrial monolayers was determined. LPS was added on apical side of HIV-1 exposed and control monolayers and one hour later basolateral supernatants were collected and LPS leakage was measured. The LPS levels in basolateral supernatants were increased by 47.3±0.922% in HIV-1 exposed monolayers in comparison with LPS leakage in mock-treated control monolayers.

We also measured translocation of HIV-1 through the primary endometrial monolayers (Figure 10C). At various time points following HIV-1 exposure on the apical side, basolateral supernatant was collected and HIV-1 infectious viral counts were determined TZMb-1 indicator cell assay. The results are presented as percent of inoculum virus added on apical side. Infectious viral counts were seen starting at 6 hours following exposure to HIV-1 (0.03% of inoculum) and infectious virus continued to accumulate in the basolateral compartment (0.08% of inoculum) up to 48 hours time, which was the last time point examined.

## Discussion

To summarize, we were able to demonstrate that exposure to HIV-1 directly decreased the transepithelial resistance across intestinal and genital epithelial monolayers. The reduction in TER correlated with significant decrease in tight junction protein expression and increased permeability, indicating functional impairment of the barrier. The effect was specific for HIV-1 and reached significant levels within 2–4 hours following HIV-1 exposure. Similar reduction in tight junction functioning was observed following treatment of ECs with HIV-1 envelope protein gp120 but not tat, a regulatory protein. Neutralization of gp120 and exposure to an Env^−^ HIV significantly abrogated the impairment of epithelial barrier, indicating that the effect was mediated by HIV-1 envelope glycoprotein. We further determined that exposure of the epithelial monolayers to HIV-1 led to enhanced production of a number of inflammatory cytokines, including TNF-α, by both intestinal and genital epithelial cells. When epithelial cells were exposed to HIV-1 in presence of anti-TNF antibody, there was no significant decrease in TER, indicating that TNF played a major role in impairing the barrier functions. In experiments designed to determine whether the disruption of epithelial barrier function could be directly associated with microbial leakage across the mucosa, we found evidence for small but significant bacterial and viral translocation across epithelial monolayers following HIV-1 exposure.

To the best of our knowledge, this is the first study to demonstrate that HIV-1 can directly disrupt mucosal epithelial barrier functions that can lead to enhanced microbial translocation. Previous clinical studies have documented that in HIV-1 infected patients intestinal permeability is altered, characterized by diarrhea-induction [15]–[17] . A recent study showed impairment of barrier function in intestinal biopsies of HAART-naïve patients compared to those on HAART treatment [14]. Increased production of cytokines IL-2, IL-4, IL-5 and TNF-α was found in supernatants of cultured intestinal biopsies in this study. Their conclusion was that following infection, HIV replication in target cells leads to local increase of inflammatory cytokines in the intestinal mucosa, which induce barrier impairment. This supports previous studies where PBMCs co-cultured with HIV-infected macrophages resulted in increased production of a number of cytokines, including TNF-α, IL-1β, IFN-α and IFN-γ which were shown to compromise epithelial barrier function [34]. The prevailing opinion from these studies is that the effect on epithelial barrier is likely mediated via immune cell activation due to viral replication [14],[25]. Of note are other studies that were unable to show that mononuclear cells isolated from colon of infected patients produce increased amount of cytokines [35],[36]. Thus far the cellular source of inflammatory cytokines that could lead to barrier disruption in HIV infected patients remains controversial [20]. Based on our results, we would like to propose that the primary sources of the inflammatory cytokines that disrupt the mucosal barrier are the epithelial cells themselves. Our studies demonstrate that epithelial cells respond directly and rapidly to HIV envelope glycoprotein by production of increased levels of cytokines which lead to loss of barrier functions, rather than an indirect effect mediated by immune cells following HIV replication. This provides an alternate and more direct explanation as to why decrease in viral load following HAART treatment restores intestinal barrier functions [14]. Our results that demonstrate that barrier dysfunction can allow bacterial translocation could also provide explanation for increased levels of immune activation during acute infection, an observation noted in a previous study which examined immune activation following HIV infection in North American cohorts [25]. The mechanism demonstrated in the present study does not exclude the possibility that cytokines released from immune cells in the HIV-infected intestines could also contribute to further disruption of the barrier, more likely in the chronic phase of the infection.

That viral exposure could directly lead to compromised barrier function has been shown before [6],[8],[9]. Many other viruses and even bacteria have been shown to directly compromise both epithelial and endothelial barrier integrity. Astrovirus, a single stranded RNA virus and a causative organism of common diarrhea was recently shown to increase epithelial barrier permeability in Caco-2 intestinal cells, modulated by its capsid protein, independent of viral replication [6]. Coxsackievirus has also been shown to directly compromise endothelial tight junctions [8]. Previous studies have shown that HIV-1 infection can compromise the blood-brain barrier thereby leading to progression of HIV-1 encephalitis (reviewed in [37]). The functioning of the tight junctions between endothelial cells, that form the blood-brain barrier, is quite similar to those present between mucosal epithelial cells. However, the mechanism elucidated by these studies was not a direct effect of HIV, but facilitated by production of TNF-α during chronic infection that mediated opening of paracellular route in endothelial lining, for viral entry into the brain. Interestingly, a recent study elucidated that HIV-1 tat protein can directly compromise the retinal epithelial barrier function [38]. Despite this evidence, no studies have so far examined the direct effect of HIV-1 exposure on mucosal epithelium. Our results show that increased permeability is mediated directly by HIV viral envelope glycoprotein. Further, given that significant disruption of tight junction proteins and decreased TERs occurred following treatment with UV inactivated virus, this phenomenon is independent of viral replication. Whether HIV-1 entry is required for the epithelial cell response is currently being examined.

In our study, both the intestinal cell line and primary genital epithelial cells showed similar response to different strains of HIV-1: disruption of tight junctions, and increased permeability. However, we found the profile of cytokines produced constitutively by intestinal and genital epithelial cells was quite distinct. While the intestinal cell line T84 did not constitutively produce TNF-α, IL-6, IL-8 and MCP-1, there was significant induction of these cytokines following HIV-1 exposure. Primary genital epithelial cultures, on the other hand, constitutively produced TNF-α, IL-6, IL-8 and MCP-1 and production of TNF-α and IL-6 was significantly upregulated following HIV-1 exposure. Both types of ECs secreted minimal levels of IL-10 and IL-1β which was upregulated following HIV-1 exposure only in primary genital epithelial cells. The differences in the constitutive cytokine profile between genital and intestinal epithelial cells could be due to distinct characteristics of primary cells compared to cell lines. Alternatively, intestinal epithelial cells are likely to be more quiescent in terms of baseline cytokine production given their microenvironment where a variety of commensal organisms are always present in the lumen [39]. In comparison, upper genital tract epithelium exist in a relatively sterile environment and are known to actively secrete an array of cytokines [2]. Nevertheless, following exposure to HIV-1 both types of ECs responded with enhanced induction of inflammatory cytokines that mediated disruption of tight junctions. This indicates that as long as the viral load and exposure times are sufficient, HIV can likely disrupt any mucosal barrier in the body, independent of infection and replication.

Among the cytokines that were upregulated, the direct effect of TNF-α on disruption of intestinal epithelial tight junction and increased permeability has been extensively characterized [33]. TNF-induced increase in permeability of Caco-2 cells is known to be mediated by NF-kB activation that downregulates ZO-1 protein expression [40]. ZO-1 proteins are integral part of the tight junction assembly and function as a scaffolding protein critical in maintaining the integrity of the tight junctions. The results from ZO-1 quantification (Figure 4) indicate that the disruption of tight junctions following HIV-1 exposure likely happens in two stages: initially there may be a displacement of ZO-1 that leads to disruption of tight junction integrity followed by marked reduction in the amount of ZO-1 and other tight junction proteins due to decreased transcription. Thus, TNF-α produced by the ECs in response to HIV-1 envelope glycoprotein could induce NF-kB activation and subsequent downregulation of tight junction proteins, including ZO-1. Our ongoing studies show that NF-kB translocation occurs within 1 hour of HIV-1 exposure (Nazli and Kaushic, unpublished). Whether there are distinct steps in this process that have discrete mechanisms is currently under investigation. Regardless of the detailed mechanism, the outcome of tight junction disruption is decrease in TER and leakage across the epithelial barrier.

The finding that disruption of barrier function can result in small but significant amount of both viral and bacterial translocation across ECs following exposure to HIV-1 has profound implications. Although previous studies have demonstrated presence of LPS in serum of HIV infected patients and correlated it with immune activation in North American cohorts, the inference that microbial flora in the intestines was the source of LPS was indirect [25]. Our studies provide direct evidence that both bacteria and virus present on the apical side of mucosal epithelial cells during HIV-1 exposure could leak through because of the impairment of epithelial tight junctions and increased permeability. This could allow HIV-1 access to target cells located in the lamina propria of the mucosa as well allow bacterial translocation that could cause local immune activation.

While the viral-epithelial interactions described here are novel, further investigation is needed to determine what role increased barrier permeability plays in initiating HIV-1 infection. HIV-1 transmission across intestinal and genital mucosa occurs predominantly via infected semen; currently the role of seminal plasma in HIV-1 transmission is far from clear. Recent studies indicate that seminal plasma can lead to inflammatory responses and facilitate HIV-1 transmission [41]–[43]. However, seminal plasma components such as TGF-β and HGF also enhance epithelial barrier functions [44]. Further, given the low efficiency of viral translocation seen in the ex-vivo model described here, the ability of HIV-1 to cross over in significant numbers, in vivo, would be depend presence of high viral load in the semen, most likely in acute phase of infection. While plasma and semen loads show overall correlation, compartmentalization between genital and blood viral loads is well recognized and more recent studies show that seminal plasma viral load can persist following treatment with HAART [45]–[47]. While the results from the present study elucidate a new mechanism that could lead to viral translocation across the epithelial barrier, more information is needed to understand how other factors like seminal plasma, stage of the infection and viral load may influence any viral leakage across the mucosal barrier. If under physiological conditions, viral leakage does occur because of barrier disruption, it could play a critical role in initiation of infection, especially in the presence of existing inflammation from other viral or bacterial co-infections [48].

In conclusion, the current study provides evidence for the first time that HIV-1 exposure at the mucosal surface leads to direct response by the mucosal epithelium, seen by production of inflammatory cytokines. This response is rapid, independent of viral infection and likely plays a key role in initiation of mucosal damage. This information will be critical for strategies to target control of mucosal damage.

## Methods

### Primary genital epithelial and intestinal cell line cultures

Reproductive tract tissues were obtained from women aged 30–59 years (mean age 42.9+7.2) undergoing hysterectomy for benign gynecological reasons at Hamilton Health Sciences Hospital. Written informed consent was obtained from all patients, with the approval of Hamilton Health Sciences Research Ethics Board. The most common reasons for surgery were uterine fibroids and heavy bleeding. Tissues were first examined by pathologists and if they were deemed free from any malignant or other clinically observed disease, coin-sized pieces were collected for further processing.

Detailed protocol for isolation and culture of genital epithelial cells (GEC) has been described previously [49]. Briefly, endometrial and cervical tissues were obtained from women undergoing hysterectomy and minced into small pieces and digested in an enzyme mixture for 1 hour at 37°C. Epithelial cells (EC) were isolated by a series of separations through nylon mesh filters of different pore sizes. EC were grown onto Matrigel™ (Becton, Dickinson and Company) coated, 0.4-µm pore-size polycarbonate membrane tissue culture inserts (BD Falcon, Mississauga, Canada) with primary tissue culture medium (DMEM/F12; Invitrogen, Canada) supplemented with 10 µM HEPES (Invitrogen, Canada), 2 µM l-glutamine (Invitrogen, Canada), 100 units/ml penicillin/streptomycin (Sigma–Aldrich, Oakville, Canada), 2.5% Nu Serum culture supplement (Becton, Dickinson and company, Franklin Lakes, USA), and 2.5% Hyclone defined fetal bovine serum (Hyclone, Logan, USA). Polarized monolayers were formed within 5–7 days. The purity of GEC monolayers was between 95% and 98%. There was no trace of any hematopoietic cells in the confluent monolayers. The methodology used for monitoring the purity of the epithelial monolayers and absence of CD45 staining in confluent cultures has been described in detail before [49].

The human colon-derived crypt-like T84 epithelial cell line was maintained and cultured as described previously [50]. Briefly T84 intestinal cells were grown and maintained in a 1∶1 (vol/vol) mixture of Dulbecco's modified Eagle's medium and Ham's F-12 medium, supplemented with 10% fetal bovine serum, 1.5% HEPES, and 2% penicillin-streptomycin (Life Technologies, Grand Island, NY) at 37°C in 5% CO_2_. T84 cells were seeded onto filter supports (0.5×10^6^ cells/well, 0.4-µm pore-size polycarbonate membrane tissue culture inserts, BD Falcon, Mississauga, Canada) and grown for approximately 5–6 days till they reached confluency. The confluency of EC cultures and T84 monolayers was monitored microscopically and by trans-epithelial resistance (TER) across monolayers grown on cell culture inserts, using a volt ohm meter (EVOM; World Precision Instruments, Sarasota, FL, USA). Epithelial monolayers showing TER values higher than 1000 Ω/cm^2^ were considered completely confluent and used for further experiments.

### Virus strains, propagation and infection

HIV-1 R5 and X4 -tropic laboratory strains were prepared by one of two methods. R5-tropic ADA and X4-tropic laboratory strain IIIB viral stocks were prepared by infection of adherent monocytes from human PBMCs (ADA) or from chronically infected H9 cell line (IIIB), followed by virus concentration by Amicon Ultra-15 filtration system (Millipore, Billerica, US). Virus stock preparations were checked for possible contamination by cellular factors by multiplex bead-based sandwich immunoassay (Luminex Corporation, Austin, TX, USA). TNF-α, IL-6, IL-8, MCP-1, MIP-1α, MIP-1β, RANTES, IL-1α, IL-1β were not detected in any viral stock (standard range of detection limit for different factors: 0.1–4.5 pg/ml). Laboratory strains of HIV-1 virus were also prepared by ultracentrifugation method. HIV-1 R5 laboratory strains ADA, Bal, and X4 strains IIIB, MN and NL4-3 and four clinical strains 11242 (dual), 11249 (R5), 4648 (R5), 7681 (X4) (Dr. Donald R. Branch, University of Toronto) were prepared in human PBMC preparations and concentrated by ultracentrifugation over 20% sucrose for 1 hour at 19,000 rpm (33,000g). All HIV-1 stocks were titered for infectious viral count/ml by TZMb-1 indicator cell assay as described previously [51]. TZMb-1 assay is based on infection of Hela cell line that has been stably transfected with CD4, CXCR4 and CCR5 receptors for HIV-1 attachment. The cell line also carries two indicator systems (β-galactosidase and luciferase systems) under the influence of HIV-1 promoter. HIV-1 infection is detected by staining the cells for β-galactosidase activity resulting in cells turning blue, indicative of HIV-1 replication or alternately by detection of luciferase activity. Infectious viral units/ml  =  infectious viral counts indicated by number of blue cells per well X dilution factor/ml.

For HIV-1 exposure, primary epithelial cells, isolated from human female genital tract tissues or intestinal T84 cells were grown to confluence. Epithelial cell cultures were exposed apically or basolaterally to HIV-1 virus (10^5^ infectious viral units/well/100µl, final concentration 10^6^ infectious viral units/ml), corresponding to MOI of 1.0 or other viral doses as mentioned in individual experiments. The p24 values corresponding to this standard concentration of virus (10^6^ infectious viral units/ml) varied, depending on the viral strain, as determined by p24 ELISA (Zeptometrix Corp., Buffalo, NY, USA); it corresponded to p24 concentration between 0.7–1110 ng/ml for X4 tropic lab strains and between 0.3–790 ng/ml for R5 tropic viruses. For clinical strains p24 concentrations used for HIV exposures were between 49–773 ng/ml. Mock infection controls included exposure to same volume of media without HIV-1 (media control, mock) or exposure to same volume of virus (and gp120) free supernatant from PBMC (for R5 HIV-1) or H-9 (X4 HIV-1) cell line cultures (R5 and X4 controls).

Env-defective mutant, Env^−^ (kind gift of D. Johnson, NCI) was on an NL4-3 backbone (X4-tropic HIV-1 laboratory strain) and was compared to wildtype NL4-3 for its effect on epithelial cell permeability [30]. T84 intestinal epithelial monolayers were exposed to wildtype (10^6^ infectious units/ml, p24 79ng/ml) or Env^−^ NL4-3 (p24, 79ng/ml) and TER were measured prior to and 24 hours post-exposure. Monolayers were fixed for ZO-1 staining.

### UV inactivation of HIV

HIV-1 R5-tropic strain ADA and X4-tropic strain IIIB were inactivated by UV exposure. 10^6^ infectious units/ml of virus was subjected to 25–100mJ/cm^2^ UV with a UV cross-linker (Fisher Scientific, USA). UV inactivation of virus was confirmed by titration on TZMb-1 cells.

### Gp120 and Tat treatments

HIV-1 proteins gp120 envelope protein and soluble Tat protein were obtained from NIH AIDS Research & Reference Reagent Program. Epithelial cell cultures were treated with HIV-1 viral proteins gp120 (0.8nM, 0.1 µg/ml) or Tat (100 nM, 1.4ug/ml). A range of Gp120 concentration (50ng–1µg/ml) was tried based on those used in previous studies [28]. Concentration of tat was consistent with that used in other studies for cultured brain endothelial cells and corneal epithelial cells [29],[38]. HIV-1 proteins were allowed to interact with the epithelial cells for 24 hours at 37°C.

### Gp120 neutralization assay

To test the role of gp120, HIV-1 IIIB was incubated at 37C with a recombinant human monoclonal neutralizing antibody against HIV-1 gp120 (IgG1, clone 2G12, Polymun Scientific, Austria) at a concentration of 35µg/ml or an isotype control antibody (Southern Biotechnology, Birmingham, USA) at same concentration for 1 hour. TERs were measured prior to and post-exposure.

### Quantitative real-time reverse transcriptase polymerase chain reaction of tight junction proteins

Quantitation of tight junction gene expression in epithelial cells post-HIV-1 exposure and comparison with unexposed control epithelial cells was done by real time quantitative reverse-transcriptase polymerase chain reaction (qRT-PCR) with Syber Green. The tight junction genes examined were Claudin 1, 2, 3, 4, 5, ZO-1, and Occludin. ECs were lysed by Trizol reagent, total RNA was extracted by RNeasy mini kit (Qiagen Inc., ON, Canada) and treated with DNase column (RNase-free DNase set, Qiagen Inc., ON, Canada) to remove DNA contamination. The cDNA was synthesized by qScript™ cDNA supermix (Quanta Bioscience Inc., Gaithersburg, MD, US) according to manufacturer's protocol. Real-time PCR was performed for each tight junction gene mRNA and GAPDH (internal control) in AB7700 SDS V1.7 (Applied Biosystems, Foster City, CA) with the program: 50°C 2 min, 94°C for 10 minutes and 40 cycles at 94°C for 15 s and 60°C for 1 minute. To validate the quantitative real-time RT-PCR protocol, melting curve analysis was performed to check for the absence of primer dimers. The sequence of primers targeting tight junction genes was taken from published studies (Table 1). The quantitative PCR data was analyzed using the comparative CT method [52]. Briefly the difference in cycle times, ΔCT, was determined as the difference between the tested gene and the reference house keeping gene GAPDH. ΔΔCT was obtained by finding the difference between exposed and mock-treatment groups for each gene. The fold change was calculated as FC = 2^−ΔΔCT^ and results were expressed as fold decrease following HIV exposure compared to mock-treated control cultures.

### Immunofluorescent staining for tight junction proteins

Following treatment, EC monolayers were fixed in 4% Paraformaldehyde, permeabilized with 0.1% Triton X-100 (Mallinckrodt Inc., Paris, KY), and blocked for 30 minutes in blocking solution (5% bovine serum albumin and 5% goat serum (Sigma-Aldrich, ON, Canada) in 0.1% Triton X-100]. Primary antibodies (rabbit anti-human claudin-2, rabbit anti-human Occludin, or rabbit anti-human ZO-1 from Zymed Laboratories, CA, USA) were diluted (2 µg/ml) in blocking solution and incubated with monolayers for 1 hour at room temperature. Normal rabbit serum was used as a negative control to check the specificity of primary antibodies. Following incubation with primary antibodies the monolayers were washed with PBS and secondary antibody, Alexa Fluor 488 goat anti-rabbit IgG (1.5 µg/ml, Molecular Probes, Eugene, OR) was added for 1 hour at room temperature. Nuclear counterstaining was done with Propidium Iodide (500nM, Molecular Probes, Eugene, OR). After extensive washing, filters were excised from the polystyrene inserts and mounted on glass slides in mounting medium (Vectashield mounting medium, Vector Lab, CA, USA). All samples were imaged on an inverted confocal laser-scanning microscope (LSM 510, Zeiss, Germany) using standard operating conditions (63× objective, optical laser thickness of 1µm, image dimension of 512×512, lasers: argon (450nm) and HeNe (543nm) for ZO-1 and nuclear staining, respectively. For each experiment, confocal microscope settings for image acquisition and processing were identical between control and treated monolayers and 3 separate, random images were acquired and analyzed for each experimental condition. Each experiment was repeated at least 3 times. Monolayers were scanned in an apical to basolateral sequence and sequential image sets were analyzed by image analysis software (Image J, NIH) to measure the areas of both fluorescently stained ZO-1 and cellular nuclei. Images are presented as either *en face* to illustrate the distribution of tight junction protein immunoreactivity or as a composite *Z*-stack reconstruction, which shows the monolayer in transverse profile with the basally located nuclei identified by propidium iodide staining (red) and tight junction proteins by fluorescein isothiocyanate labeled secondary antibodies (green). For Figure 4A–C, optical sections (XY planes) through the apical regions of monolayers were stacked to represent complete tight junction ZO-1 staining distribution in order to make direction comparison between control and experimental counterparts.

### MTT viability assay

MTT assay was used to determine viability of HIV-1 exposed monolayers and compared to unexposed control monolayers. The assay was performed according to manufactures instructions (Biotium Inc., CA, USA). Briefly, human primary endometrial epithelial cells and T84 intestinal epithelial cells were seeded on 96-well plates at a density of 10^3^ cells/well and allowed to attach to the plate and grown for 5 days. Triplicate wells were treated with media or exposed with laboratory strains of HIV-1 (10^4^ infectious viral units/ml, MOI 1∶1) in 100 µl quantity. After 24 hours incubation, 10 µl of MTT solution was added and incubated for 4h at 37°C. After incubation, the medium was discarded and the purple blue sediment was dissolved in 200 µl DMSO. The relative optical density (OD)/well were determined at a test wavelength of 570 nm in a ELISA reader using a 630 nm reference wavelength. The MTT assay is based on the cleavage of the yellow tetrazolium salt (MTT) to purple formazan by metabolically active cells, based on their mitochondrial activity. Cell viability was expressed as a percentage of untreated cells, which served as a negative control group and was designated 100%; the results are expressed as % of negative control. All assays were performed in triplicate.

### Blue Dextran leakage assay

Blue Dextran dye was dissolved in primary medium (2.3 mg/ml, [26]) and added to the apical surface of confluent epithelial cell monolayers grown on 0.4µm pore size culture inserts. At various time intervals, post-HIV-1 exposure, 50ml of basolateral medium was sampled and replaced by equal volume of primary growth media. Blue Dextran dye in basolateral samples was measured using a microplate reader (Safire, tecan, NC, USA) at 610nm and the optical density was expressed as a % of density of dye added to the apical medium at the beginning of experiment (Time “0”).

### Cytokine analysis

Apical and basolateral supernatants were analyzed for multiple cytokines using the Luminex multianalyte technology (Luminex Corporation, Austin, TX, USA) as described before [53]. Multiplex bead-based sandwich immunoassay kits (Upstate Biotech, Millipore, MA, USA) were used to measure levels of IL-1β, IL-6, IL-8, IL-10, MCP-1 and TNF-α, as per the manufacturer's instructions. Primary endometrial EC and T84 monolayers were exposed to HIV-1 (ADA strain, 10^6^ infectious viral units/ml) and apical and basolateral supernatants were collected after 24 hours. Minimum detection limit for the cytokines were 0.1 pg/ml for TNF-α, 0.2 pg/ml for IL-8, 0.3 pg/ml for IL-6 and IL-10, 0.4 pg/ml for IL-1β, 0.9 pg/ml for MCP-1. Levels detected at or below this limit were considered and reported as undetectable.

### TNF-α neutralization assay

Epithelial cells were grown to confluence and treated with TNF-α (20ng/ml) or HIV-1 (ADA, 10^6^ infectious viral units /ml ) for 24 hours. To test the role of TNF-α, mouse anti-human TNF-α neutralizing antibody (25 µg/ml) (R&D Systems, USA) or normal mouse serum (25 µg/ml) was added to confluent monolayers for 1 hour at 37C prior to treatment with TNF-α (20ng/ml) or HIV-1. Barrier function was determined by TER measurements before and after treatment.

### Bacterial and HIV-1 translocation

For bacterial translocation experiments, non-pathogenic *E. coli* strain HB101 was grown and cultured in Luria-Bertani (LB) broth (Invitrogen, Canada). T84 cells were grown to confluence on 3.0-µm-pore-size filters (BD Falcon, Canada), transferred to antibiotic-free Hanks solution, and treated with TNF-α (20ng/ml), *E.coli* (10^8^ CFU/ml), HIV-1 (10^6^ infectious virus units/ml) for 6h, HIV-1 (10^6^ infectious viral units/ml) for 24h, TNF-α+*E.coli*, HIV-1 +* E.coli* at the same time for 6h and HIV-1 for 24h + *E.coli* for 6h. Some wells were left untreated as negative controls. TER was measured before and after treatment and basolateral supernatants were collected 6 hours after the addition of *E. Coli* to detect bacterial translocation to the basolateral side. The supernatants were diluted and plated on LB agar and incubated for 24h followed by enumeration of bacterial colony counts.

For viral translocation, HIV-1 was added to the apical surface of confluent EC monolayers at a concentration of 10^5^ infectious viral units/well and basolateral supernatants were collected at different time intervals. Viral counts were determined using TZMb-1 indicator cell assay.

For assessment of LPS leakage, LPS (100ng/ml; from *E.coli* O26:B6; Sigma-Aldrich, MO, USA) was added to the apical surface of confluent EC monolayers, 24h post-exposure to HIV and compared with unexposed controls. Basolateral supernatants were collected 1 hour after addition of LPS and LPS leakage was measured by measuring LPS levels in the basolateral supernatants by Pyrochrome LPS detection kit (Cape Cod incorporated, MA, USA) according to the manufacturer's instructions.

### Statistical analysis

GraphPad Prism Version 4 (GraphPad Software, San Diego, CA) was used to compare three or more means by 2 way analysis of variance (ANOVA). When an overall statistically significant difference was seen, post-tests were performed to compare pairs of treatments, using the Bonferroni method to adjust the *p*-value for multiple comparisons. An alpha value of 0.05 was set for statistical significance. *p*-Values for each analysis are indicated in figure legends.

### Accession numbers of genes and proteins

TNF-a (NCBI Accession number AAD18091), IL-8 (NCBI Accession number CAA77745), IL-6 (NCBI Accession number AAD13886), IL-10 (NCBI Accession number AAA63207), IL-1b (NCBI Accession number AAC03536), MCP-1 (NCBI Accession number AABB29926). ZO-1 (GeneBank Accession number NM_003257), Occludin (GeneBank Accession number NM_002538), Claudin-1 (Genebank Accession number NM_021101, Claudin-2 (Genebank Accession number NM_020384), Claudin-3 (Genebank Accession number NM_001306), Claudin-4 (Genebank Accession number NM_001305), Claudin-5 (Genebank Accession number NM_003277), GAPDH (Genebank Accession number NM_002046).

## References

[1] Shattock RJ, Haynes BF, Pulendran B, Flores J, Esparza J (2008). Improving defences at the portal of HIV entry: mucosal and innate immunity.. PLoS Med.

[2] Wira CR, Grant-Tschudy KS, Crane-Godreau MA (2005). Epithelial cells in the female reproductive tract: a central role as sentinels of immune protection.. Am J Reprod Immunol.

[3] Vroling AB, Fokkens WJ, van Drunen CM (2008). How epithelial cells detect danger: aiding the immune response.. Allergy.

[4] Yu QH, Yang Q (2009). Diversity of tight junctions (TJs) between gastrointestinal epithelial cells and their function in maintaining the mucosal barrier.. Cell Biol Int.

[5] Shacklett BL, Critchfield JW, Ferre AL, Hayes TL (2009). Mucosal T-cell responses to HIV: responding at the front lines.. J Intern Med.

[6] Moser LA, Carter M, Schultz-Cherry S (2007). Astrovirus increases epithelial barrier permeability independently of viral replication.. J Virol.

[7] Chen ML, Ge Z, Fox JG, Schauer DB (2006). Disruption of tight junctions and induction of proinflammatory cytokine responses in colonic epithelial cells by Campylobacter jejuni.. Infect Immun.

[8] Ju Y, Wang T, Li Y, Xin W, Wang S (2007). Coxsackievirus B3 affects endothelial tight junctions: possible relationship to ZO-1 and F-actin, as well as p38 MAPK activity.. Cell Biol Int.

[9] Nava P, Lopez S, Arias CF, Islas S, Gonzalez-Mariscal L (2004). The rotavirus surface protein VP8 modulates the gate and fence function of tight junctions in epithelial cells.. J Cell Sci.

[10] Sanders DS (2005). Mucosal integrity and barrier function in the pathogenesis of early lesions in Crohn's disease.. J Clin Pathol.

[11] Brenchley JM, Douek DC (2008). HIV infection and the gastrointestinal immune system.. Mucosal Immunol.

[12] Hladik F, Hope TJ (2009). HIV infection of the genital mucosa in women.. Curr HIV/AIDS Rep.

[13] Hladik F, McElrath MJ (2008). Setting the stage: host invasion by HIV.. Nat Rev Immunol.

[14] Epple HJ, Schneider T, Troeger H, Kunkel D, Allers K (2009). Impairment of the intestinal barrier is evident in untreated but absent in suppressively treated HIV-infected patients.. Gut.

[15] Lim SG, Menzies IS, Lee CA, Johnson MA, Pounder RE (1993). Intestinal permeability and function in patients infected with human immunodeficiency virus. A comparison with coeliac disease.. Scand J Gastroenterol.

[16] Keating J, Bjarnason I, Somasundaram S, Macpherson A, Francis N (1995). Intestinal absorptive capacity, intestinal permeability and jejunal histology in HIV and their relation to diarrhoea.. Gut.

[17] Sharpstone D, Neild P, Crane R, Taylor C, Hodgson C (1999). Small intestinal transit, absorption, and permeability in patients with AIDS with and without diarrhoea.. Gut.

[18] Stockmann M, Schmitz H, Fromm M, Schmidt W, Pauli G (2000). Mechanisms of epithelial barrier impairment in HIV infection.. Ann N Y Acad Sci.

[19] Stockmann M, Fromm M, Schmitz H, Schmidt W, Riecken EO (1998). Duodenal biopsies of HIV-infected patients with diarrhoea exhibit epithelial barrier defects but no active secretion.. Aids.

[20] Brenchley JM, Douek DC (2008). The mucosal barrier and immune activation in HIV pathogenesis.. Curr Opin HIV AIDS.

[21] Redd AD, Dabitao D, Bream JH, Charvat B, Laeyendecker O (2009). Microbial translocation, the innate cytokine response, and HIV-1 disease progression in Africa.. Proc Natl Acad Sci U S A.

[22] Brenchley J, Price D, Douek D (2006). HIV Disease: Fallout from a mucosal catastrophe?. Nat Immunol.

[23] Hazenberg MD, Otto SA, van Benthem BH, Roos MT, Coutinho RA (2003). Persistent immune activation in HIV-1 infection is associated with progression to AIDS.. Aids.

[24] Giorgi JV, Hultin LE, McKeating JA, Johnson TD, Owens B (1999). Shorter survival in advanced human immunodeficiency virus type 1 infection is more closely associated with T lymphocyte activation than with plasma virus burden or virus chemokine coreceptor usage.. J Infect Dis.

[25] Brenchley JM, Price DA, Schacker TW, Asher TE, Silvestri G (2006). Microbial translocation is a cause of systemic immune activation in chronic HIV infection.. Nat Med.

[26] Velarde G, Ait-Aissa S, Gillet C, Rogerieux F, Lambre C (1999). Use of Transepithelial Electrical resistance in the study of Pentachlorophenol Toxicity.. Toxicology in vitro.

[27] Annunziata P (2003). Blood-brain barrier changes during invasion of the central nervous system by HIV-1. Old and new insights into the mechanism.. J Neurol.

[28] Kanmogne GD, Schall K, Leibhart J, Knipe B, Gendelman HE (2007). HIV-1 gp120 compromises blood-brain barrier integrity and enhances monocyte migration across blood-brain barrier: implication for viral neuropathogenesis.. J Cereb Blood Flow Metab.

[29] Pu H, Tian J, Andras IE, Hayashi K, Flora G (2005). HIV-1 Tat protein-induced alterations of ZO-1 expression are mediated by redox-regulated ERK 1/2 activation.. J Cereb Blood Flow Metab.

[30] Ott DE, Chertova EN, Busch LK, Coren LV, Gagliardi TD (1999). Mutational analysis of the hydrophobic tail of the human immunodeficiency virus type 1 p6(Gag) protein produces a mutant that fails to package its envelope protein.. J Virol.

[31] Fichorova RN, Desai PJ, Gibson FCI, Genco CA (2001). Distinct proinflammatory host responses to Neisseria gonorrhoeae infection in immortalized human cervical and vaginal epithelial cells.. Infec Imm.

[32] Al-Sadi R, Boivin M, Ma T (2009). Mechanism of cytokine modulation of epithelial tight junction barrier.. Front Biosci.

[33] Ye D, Ma I, Ma TY (2006). Molecular mechanism of tumor necrosis factor-alpha modulation of intestinal epithelial tight junction barrier.. Am J Physiol Gastrointest Liver Physiol.

[34] Schmitz H, Rokos K, Florian P, Gitter AH, Fromm M (2002). Supernatants of HIV-infected immune cells affect barrier function of human HT-29/B6 intestinal epithelial cells.. AIDS.

[35] Steffen M, Reinecker HC, Petersen J, Doehn C, Pfluger I (1993). Differences in cytokine secretion by intestinal mononuclear cells, peripheral blood monocytes and alveolar macrophages from HIV-infected patients.. Clin Exp Immunol.

[36] Snijders F, van Deventer SJ, Bartelsman JF, den Otter P, Jansen J (1995). Diarrhoea in HIV-infected patients: no evidence of cytokine-mediated inflammation in jejunal mucosa.. Aids.

[37] Brabers NA, Nottet HS (2006). Role of the pro-inflammatory cytokines TNF-alpha and IL-1beta in HIV-associated dementia.. Eur J Clin Invest.

[38] Bai L, Zhang Z, Zhang H, Li X, Yu Q (2008). HIV-1 Tat protein alter the tight junction integrity and function of retinal pigment epithelium: an in vitro study.. BMC Infect Dis.

[39] Kagnoff MF, Eckmann L (1997). Epithelial cells as sensors for microbial infection.. J Clin Invest.

[40] Ma TY, Iwamoto GK, Hoa NT, Akotia V, Pedram A (2004). TNF-alpha-induced increase in intestinal epithelial tight junction permeability requires NF-kappa B activation.. Am J Physiol Gastrointest Liver Physiol.

[41] Sharkey DJ, Macpherson AM, Tremellen KP, Robertson SA (2007). Seminal plasma differentially regulates inflammatory cytokine gene expression in human cervical and vaginal epithelial cells.. Mol Hum Reprod.

[42] Gutsche S, von Wolff M, Strowitzki T, Thaler CJ (2003). Seminal plasma induces mRNA expression of IL-1beta, IL-6 and LIF in endometrial epithelial cells in vitro.. Mol Hum Reprod.

[43] Munch J, Rucker E, Standker L, Adermann K, Goffinet C (2007). Semen-derived amyloid fibrils drastically enhance HIV infection.. Cell.

[44] Planchon S, Fiocchi C, Takafuji V, Roche JK (1999). Transforming growth factor-beta1 preserves epithelial barrier function: identification of receptors, biochemical intermediates, and cytokine antagonists.. J Cell Physiol.

[45] Pilcher CD, Joaki G, Hoffman IF, Martinson FE, Mapanje C (2007). Amplified transmission of HIV-1: comparison of HIV-1 concentrations in semen and blood during acute and chronic infection.. Aids.

[46] Tachet A, Dulioust E, Salmon D, De Almeida M, Rivalland S (1999). Detection and quantification of HIV-1 in semen: identification of a subpopulation of men at high potential risk of viral sexual transmission.. Aids.

[47] Sheth PM, Kovacs C, Kemal KS, Jones RB, Raboud JM (2009). Persistent HIV RNA shedding in semen despite effective antiretroviral therapy.. Aids.

[48] Kaul R, Pettengell C, Sheth PM, Sunderji S, Biringer A (2008). The genital tract immune milieu: an important determinant of HIV susceptibility and secondary transmission.. J Reprod Immunol.

[49] MacDonald EM, Savoy A, Gillgrass A, Fernandez S, Smieja M (2007). Susceptibility of Human Female Primary Genital Epithelial Cells to Herpes Simplex Virus, Type-2 and the Effect of TLR3 Ligand and Sex Hormones on Infection.. Biol Reprod.

[50] McKay DM, Singh PK (1997). Superantigen activation of immune cells evokes epithelial (T84) transport and barrier abnormalities via IFN-gamma and TNF alpha: inhibition of increased permeability, but not diminished secretory responses by TGF-beta2.. J Immunol.

[51] Kimpton J, Emerman M (1992). Detection of replication-competent and pseudotyped human immunodeficiency virus with a sensitive cell line on the basis of activation of an integrated beta-galactosidase gene.. J Virol.

[52] Schmittgen TD, Livak KJ (2008). Analyzing real-time PCR data by the comparative C(T) method.. Nat Protoc.

[53] Nazli A, Yao XD, Smieja M, Rosenthal KL, Ashkar AA (2009). Differential induction of innate anti-viral responses by TLR ligands against Herpes simplex virus, type 2, infection in primary genital epithelium of women.. Antiviral Res.

